# Predictors of *Chlamydia Trachomatis* testing: perceived norms, susceptibility, changes in partner status, and underestimation of own risk

**DOI:** 10.1186/s12889-016-2689-6

**Published:** 2016-01-20

**Authors:** Gill A. ten Hoor, Robert A. C. Ruiter, Jan E. A. M. van Bergen, Christian J. P. A. Hoebe, Nicole H. T. M. Dukers-Muijrers, Gerjo Kok

**Affiliations:** 1 Department of Work & Social Psychology, Maastricht University, P.O. Box 616, 6200 MD Maastricht, The Netherlands; 2 STI AIDS Netherlands, Keizersgracht 390, 1016GB Amsterdam, The Netherlands; 3 Department of General Practice, AMC-University of Amsterdam, P.O. Box 19268, 1000GG Amsterdam, Netherlands; 4 Department of Sexual Health, Infectious Disease and Environmental Health, Public Health Service South Limburg, P.O. Box 2022, 6160HA Geleen, The Netherlands; 5 Department of Medical Microbiology, Maastricht University, P.O. Box 5800, 6202AZ Maastricht, The Netherlands

**Keywords:** STD, STI, Screening, Stereotypical beliefs, Risk perception, Sexual behavior

## Abstract

**Background:**

It is hard to convince people to participate in chlamydia screening programs outside the clinical setting. In two earlier studies (BMC Public Health. 2013;13:1091; J Med Internet Res. 2014;16(1):e24), we identified explicit and implicit determinants of chlamydia screening behavior and attempted, unsuccessfully, to improve participation rates by optimizing the recruitment letter. In the present study, we examined the links between a number of social-cognitive determinants (e.g., stereotypical beliefs about a person with chlamydia, intentions, changes in partner status), and self-reported chlamydia testing behavior six months after the initial study.

**Methods:**

The present study is a follow-up to our first study (T0). We assessed self-reported testing behavior 6 months after the first measure by means of an online questionnaire (T1; *N* = 269). Furthermore, at T1, we measured the social-cognitive determinants in more detail, and explored the influence of stereotypical beliefs and any changes in partner status during this six month period.

**Results:**

In total, 25 (9.1 %) of the participants tested for chlamydia at some point during the six months between baseline (T0) and follow up (T1). Testing behavior was influenced by testing intentions in combination with changes in risk behavior. The higher the participants’ own numbers of partners ever, the higher they estimated the number of partners of the stereotypical person with chlamydia. Testing intentions were most strongly predicted by perceived norms and susceptibility, and having had multiple partners in the last 6 months (R^2^ = .41).

**Conclusion:**

The most relevant determinants for testing intentions and behavior were susceptibility, subjective norms and changes in partner status. We found a systematic tendency for individuals to underestimate their own risk, especially the risk of inconsistent condom use. Future research should focus on more promising alternatives to population-based interventions, such as online interventions, screening in primary care, the rescreening of positives, and clinic-based interventions. This future research should also focus on making testing easier and reducing barriers to testing, as well as using social and sexual networks in order to reach more people.

**Electronic supplementary material:**

The online version of this article (doi:10.1186/s12889-016-2689-6) contains supplementary material, which is available to authorized users.

## Background

In the present study, we attempt to analyze which determinants are related to the low uptake of *Chlamydia trachomatis* (Ct) testing in a group of 16–30 year-old individuals drawn from the general population. It is hard to convince people to participate in Ct screening programs outside the clinical setting [1, 2]. In 2008, in three regions in the Netherlands, all 16–29-year-old citizens were given the opportunity to test for Ct free of charge. Via an invitation letter sent by the Public Health Services (PHS), they were asked to visit a website where they could request a Ct home-test package. Individuals could then use the test-kit to perform a Ct test at home and send it anonymously to a laboratory. Within two weeks, they were able to review their test results online. However, only a small minority of the individuals invited actually participated in this scheme (between 9.5 and 16.1 %) [3].

The current study is the third in a series of studies assessing Ct testing in 16–29 year olds in Limburg, the Netherlands. In our first study, we identified determinants of Ct screening behavior and reasons for non-participation [1]. In that study, 713 16–29-year-olds who had not tested in the past 6 months were asked questions about their intention to participate in Ct screening. We also measured their attitudes, subjective norms, perceived behavioral control, moral norms, susceptibility, descriptive norms, outcome expectations, and unrealistic optimism (social-cognitive determinants). Our results showed that these participants reported a very low intention to participate in the Ct screening program (*M* = 1.42 on a scale of 1–5). Intention was found to correlate positively with subjective norms, moral norms, susceptibility, descriptive norms, attitude and outcome expectations, and negatively with unrealistic optimism. Furthermore, Ct screening was associated with implicit measures of reassurance, as well as threat and annoyance, but these implicit measures were not related to intention to test. A first attempt was also made to assess the influence of two letters inviting 16–29-year-olds to test: the original PHS invitation letter and a letter that had been slightly adapted in line with Protection Motivation Theory [4]. The results showed no differences in testing intention between the two letters. However, receiving a letter had, compared to not receiving a letter, a positive effect on the participants’ evaluations and intention to request a Ct test package.

In our second study [2], the question was whether participation rates could be improved as a result of optimizing the invitation (recruitment) letter by systematically applying various behavior change theories [5]. Moreover, the theory-based invitation letter that had been used in the first study was adapted to take into account the findings derived from our first study, and tailored to the relevant determinants of testing behavior outlined in other earlier studies (see: http://dx.doi.org/10.2196/jmir.2907). This time, it was not behavioral intention, but only the behavior itself that was monitored. One of two different letters inviting individuals to participate in the Ct screening was randomly sent to all 16–29-year-old citizens of the ‘Sittard-Geleen’ municipality. Of the 9883 young people invited to participate, 11.4 % requested a test package. No significant differences were found in the number of test package requests between the two letter types. It was evident that the new letters did not improve participation rates as compared to the original letter.

One possible explanation is that a letter is not the best medium with which to reach this target group. It is also possible that our understanding of the relevant determinants influencing CT testing is not yet good enough. One possible determinant for risk perception that we did not measure in the first two studies is stereotypical beliefs about a person with Ct. Duncan et al. [6] interviewed 17 women with a current or recent diagnosis of Ct, and reported that most women in their study had not previously perceived themselves to be at risk, in large part due to the stereotypical beliefs these women held about who was “at risk” of sexually transmitted infection (e.g., “I thought people like me don’t get these kind of things”).

In the current study, we followed up on our first study (T0) by returning to a part of that sample and collecting new data six months later (T1). We assessed both actual (self-reported testing) behavior and stereotypical beliefs held by participants. We also measured changes in partner status between T0 and T1. Finally, we measured the same social-cognitive determinants as tested at T0, with some slight modifications (improvements based on the results of the first study).

## Method

This study was approved by the Research Ethics Board of the Faculty of Psychology & Neuroscience at Maastricht University. All research materials, data, syntax files, and output files are combined in a .zip archive labelled Additional file 1.

### Participants

As in the first study, participants were invited via Flycatcher, a representative online participant panel (http://www.flycatcher.eu/). Using anonymous IDs, we invited only participants who had participated in our first study regarding determinants of Ct testing (T0; *N* = 1822) six months before the start of the present study [1]. Our intention was to oversample people at high risk for Ct. All participants with an increased risk for Ct were invited (here, an increased risk was formulated as: participants who, at T0, reported that they had not tested for Ct in the six months before T0, and who reported having multiple sex partners in the past; *N* = 132). The other participants were selected at random from the rest of the individuals who had participated in the first study and who also indicated that they had not tested for Ct in the six months prior to T0 (*N* = 218). The participation rate was 80 % (*N* = 280; 185 with number of partners ≤ 1 and 95 with number of partners > 1). Due to the oversampling of high-risk participants, the sample in this study is not a representative sample of 18–29-year-old in the general population.

### Questionnaire

After giving informed consent online, participants were asked to fill out a short questionnaire. Participants were asked whether they had tested for Ct in the last 6 months (the time between the first study and the present study). Additional questions were asked about their sexual orientation (in order to exclude lesbian women because of their lower risk), about the number of sex partners ever and in the last 6 months (0; 1; 2; 3–5; 6–10; >10), and frequency of condom use in the last six months (1 = never – 5 = always).

The same questions as those used in ten Hoor et al. [1, 7, 8] (with slight modifications for improvement) were asked about the participant’s susceptibility (3 items), attitudes (5 items), outcome expectations (2 items), unrealistic optimism (3 items), social norms (3 items), descriptive norms (2 items), moral norms (2 items), perceived behavioral control (3 items), and intentions in relation to Ct (2 items), see Table 2 in http://www.biomedcentral.com/1471-2458/13/1091. Due to skewed distributions, questions regarding susceptibility and intention were now asked using a response scale with a broader range (1–9 in place of 1–5). New questions were added to existing questions measuring unrealistic optimism (i.e., Imagine someone of your age and gender: I think I did more than others to prevent chlamydia; totally disagree-totally agree), and perceived behavioral control (i.e., Imagine that you want to test for chlamydia, do you think you will manage even if you are very busy?; absolutely-absolutely not). One question regarding susceptibility (i.e., I think the chance is very small that I have gotten chlamydia in the past few years; totally disagree-totally agree) was deleted because it was very similar to the question My sexual behavior over the past few years makes it very probable that I have chlamydia.

Finally, we assessed participants’ stereotypical beliefs about a person with Ct, i.e., Imagine someone of your age who is infected with chlamydia (not necessarily a specific person). How many sex partners, do you think, would this person have had? How often, do you think, would this person have used condoms? These estimations were compared to the reports provided on their own behavior, e.g., frequency of condom use associated with a person their age infected with Ct minus frequency of their own condom use (∆). Higher scores represent an underestimation of own risk and/or an overestimation of the risk of a comparable other with Ct.

### Analyses

**Fig. 1:**
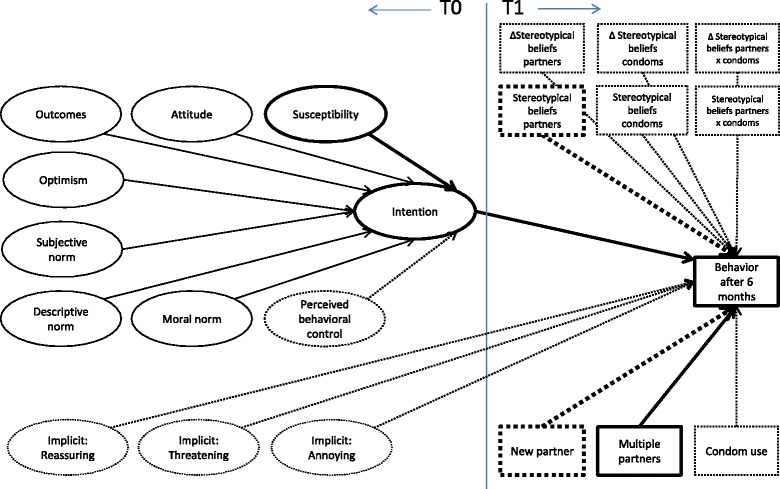
Logic Model of Predictors of Testing Intention and Behavior. No association with intention at T0 or behavior at T1. Bivariate association with intention at T0 but no multivariate association. Bivariate association with behavior at T1 but no multivariate association. Significant multivariate association with behavior at T1

IBM SPSS statistics 20 (IBM Corp. Armonk, NY, USA) was used to analyze the data. All data was recoded in such a way that a higher number reflects a higher score on the concept measured (we expected all social-cognitive determinants to positively correlate with intention to test, apart from unrealistic optimism, which we expected to negatively correlate with intention to test). In the first study, there were three different letter conditions, and no significant differences were found between them. In the current study, these three conditions did not significantly differ between high- and low-risk groups either at T0 or T1 (see Fig. 1 captions). We used the compiled data for analyses.

For the prediction regarding testing behavior, we first compared (T1) testers with non-testers on T0 intentions, social-cognitive determinants, implicit measures, as well as T1 stereotypical beliefs measures, and self-reported risk behaviors over the last six months, using independent sample T-tests. We then applied stepwise binary logistic regression to predict T1 testing behavior from all variables that showed a significant difference between testers and non-testers. See Additional file 1 for the full SPSS syntax script. For the prediction of intention at T1 from the measures at T1 we used correlation and hierarchical multiple regression analyses. See Additional file 1 for the full SPSS syntax script.

## Results

### Participants

The total number of participants was 280; five women with only female sex partners were excluded from the analyses. Moreover, six participants who reported many more (>5) partners ever at T0 than at T1 were also excluded; final *N* = 269; 190 women and 79 men. Age range was 16–30 years, *M* = 24.9.

### Low risk versus high risk

Most people with low risk at T0 (i.e., participants who, reported that they either had tested for Ct in the six months before T0, or who reported not having multiple sex partners in the past) at T0 remained low-risk at T1. Nevertheless, there were instances of high intenders that did not test (*N* = 18) and low intenders that did test (*N* = 5). From these five low intenders that did test, four reported having a new partner in the last 6 months, one reported having multiple partners in the last six months and four reported not always having used condoms in the last 6 months. When, at T0, a participant belonged to the low-risk group, at T1 that same participant might have been part of a high-risk group and vice versa (see Figure A in the Additional file 1).

### Predicting testing behavior

The first research question concerned the prediction of testing behavior after 6 months (T1) by T0 intentions, social-cognitive determinants, and implicit measures, as well as T1 stereotypical beliefs measures, and self-reported risk behaviors over the last six months. Of the 269 participants, 25 had tested in the last 6 months.

Intentions at T0 differed significantly between testers and non-testers at T1, *M*_*t*_ = 5.28 (3.21), *M*_*n*_ = 2.36 (1.95), *t* = 4.46 (*df* = 267), *p* < .001, Cohen’s *d* = 1.37. Due to the skewed distribution, we also carried out a Mann–Whitney UTest with intention (U = 1461, *p* < .001), which produced the same result.

Table 1 presents the mean differences between testers and non-testers (at T1) in terms of all potential predictors at T0. The 25 participants who had tested in the past six months had higher scores with regard to susceptibility, attitude, outcome expectations, subjective, descriptive and moral norms, and lower scores with regard to unrealistic optimism at T0. Those who had tested in the last 6 months were more likely to have had a new partner in the last six months and were more likely to have had multiple partners in the last six months (assessed at T1). Both groups scored very highly on perceived behavioral control and did not differ in this respect. There were no differences between the two groups in terms of implicit measures at T0 (see Table 1).

**Table 1:** Determinants of testing behavior

Determinant	Not tested in the last 6 months (*N* = 244) M (SD)	Tested in the last 6 months (*N* = 25) M (SD)	t	*p*
Intention at T0 (1–9)	2.36 (1.95)	5.28 (3.21)	4.46	<.001
Susceptibility at T0 (1–9)	2.69 (1.82)	4.47 (1.91)	4.61	<.001
Attitude at T0 (1–5)	2.93 (0.65)	3.30 (0.61)	2.80	.01
Outcome Expectations at T0 (1–5)	3.43 (1.11)	4.18 (0.69)	4.82	<.001
Unrealistic Optimism at T0 (1–5)	2.97 (0.85)	2.26 (0.86)	−3.96	<.001
Subjective Norms at T0 (1–5); *N* = 219/24	1.31 (0.67)	2.01 (1.21)	2.82	.01
Descriptive Norms at T0 (1–5); *N* = 163/19	2.38 (1.18)	3.11 (1.38)	2.48	.02
Moral norms at T0 (1–5)	2.32 (1.08)	3.24 (1.42)	3.14	.01
Perceived Behavioral Control at T0 (1–5)	4.41 (.079)	4.52 (0.91)	.64	.52
Implicit: Reassuring at T0; *N* = 242/25	0.29 (0.36)	0.22 (0.26)	−.95	.34
Implicit: Threatening at T0; *N* = 244/24	0.15 (0.31)	0.16 (0.38)	.17	.87
Implicit: Annoying at T0; *N* = 241/25	0.18 (0.35)	0.18 (0.30)	.002	.99
Stereotypical beliefs: Partners at T1 (1–6)	4.18 (1.20)	4.76 (0.83)	2.36	.02
Stereotypical beliefs: Condoms at T1 (1–5)	3.13 (0.98)	3.16 (0.80)	.16	.87
Stereotypical beliefs partners x condoms at T1	13.32 (5.89)	15.28 (5.45)	1.59	.11
Δ Stereotypical beliefs partners at T1	0.51 (1.33)	0.08 (1.32)	−1.54	.12
Δ Stereotypical beliefs condom use at T1	0.31 (1.19)	−0.04 (1.43)	−1.38	.17
Δ Stereotypical beliefs partners x condoms at T1	0.02 (1.87)	−0.28 (2.13)	−.77	.44
New partners last 6 m at T1 (1–2)	1.67 (0.47)	1.88 (0.33)	2.91	.01
Multiple partners 6 m at T1(1–6)	2.21 (0.64)	2.92 (1.19)	2.93	.01
Condom use last 6 m at T1 (1–5)	3.41 (1.54)	3.60 (1.26)	.70	.55

Moreover, the estimated number of partners of a stereotypical person with Ct differed between testers and non-testers: testers estimated the stereotypical person with Ct to have more partners (see Table 1). However, the higher the participants’ own numbers of partners ever, the higher they estimated the number of partners of the stereotypical person with Ct, r = .26, *p* < .001; see Tables 2 and 4.

**Table 2:** Self-reported number of partners versus estimated number of partners of stereotypical belief

Number of partners ever (range 1–6)	*N*	Stereotypical beliefs (range 1–6) *M* (*SD*)
1: 0 partners	0	-
2: 1 partners	69	3.64 (1.18)
3: 2 partners	37	3.76 (1.12)
4: 3–5 partners	86	4.29 (0.97)
5: 6–10 partners	43	4.77 (1.13)
6: > 10 partners	34	5.15 (0.89)

We predicted testing behavior at T1 as dependent variable applying stepwise Binary Logistic Regression; see Table 3 and Fig. 1.

**Table 3:** Logistic regression predicting testing in the past 6 months

Model^a^	Predictors:	Observed:	Predicted			*β/*SE/Wald
			Ct test		Percentage correct	
			No	Yes		
1.	Susceptibility T0^b^	Ct test: No	244	0	100.0	^b^-.44/.11/16.84
		Yes	25	0	0.0	
		Percentage			**90.7**	
2.	Intention T0^c^	Ct test: No	238	6	97.5	^c^-.42/.-8/28.06
		Yes	18	7	28.0	
		Percentage			**91.1**	
3.	Intention T0^d^	Ct test: No	242	2	99.2	^d^-.40/.08/22.55
						^e^.78/.25/9.97
	Multiple partners T1^e^	Yes	19	6	24.0	
		Percentage			**92.2**	

^a^Here, only the significant predictors are displayed

model 1: all social-cognitive determinants predicting of intention (i.e., susceptibility, attitude, outcome expectations, unrealistic optimism, and subjective, descriptive and moral norms at T0

model 2: intention and susceptibility

model 3: intention, multiple partners in the last six months, new partner in the last six months, and stereotypical beliefs about number of partners

b,c,d,e; same letters refer to the predictor - β/SE/Wald-value combination

Bold text: the overall percentage of correctly predicted cases per model

Of all determinants of intention (i.e., susceptibility, attitude, outcome expectations, unrealistic optimism, and subjective, descriptive and moral norms at T0), only susceptibility was a significant predictor of testing behavior in the binary logistic regression. Even though susceptibility alone predicted non-testing for all participants who did not test, the overall percentage of correctly predicted cases was only 90.7, due to the skewed distribution of testing behavior. In a second model, we tested intention (the contribution of susceptibility became non significant and was left out) as possible predictor of testing behavior. Intention contributed significantly to the prediction of testing (91.1 %). In the third step, we also included the predictors: multiple partners in the last 6 months, new partner in the last 6 months, and stereotypical beliefs about number of partners; next to intention, only multiple partners showed a significant contribution (92.2 %). The percentages refer to the percentage correctly classified.

### Determinants of testing intentions

The second research question concerned the prediction of testing intentions, all measured at T0 as well as T1.

#### Correlations and regression analyses

For all participants, except those who had tested in the last six months (whose intentions would probably have been low as they had would have recently tested and the intention refers to testing within the next 3 months), we computed correlations among the intentions and determinants (see Table 4), and performed hierarchical multiple regression analyses, predicting intentions from determinants both at T0 and at T1 (see Table 5). In the regression analyses, we only included determinants which significantly correlated with intentions (*p* < .05).

**Table 4:**
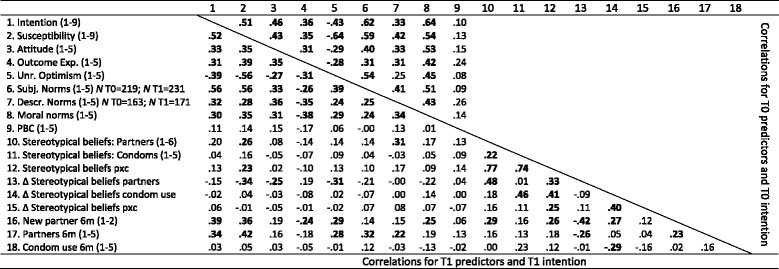
Determinants of testing intentions (*N* = 244): Correlations*; upper right part: T0; lower left part: T1

* All correlations significant at p < .001 are in bold. For T0, all *r’s* > .25 are significant at *p* < .001. For T1 all *r*’s > .21 are significant at *p* < .001

**Table 5:** Regression analyses of determinants on intentions at T1*

N = 244 (without ppn tested last 6 months)	Intention T1 M = 2.28 (1.68)							
Determinants at T1	r		beta		beta		beta	
Susceptibility	.52	***	.23	***	.24	**	.22	**
Attitude	.33	***	.06		.07		.08	
Outcomes	.31	***	.04		.05		.05	
Unrealistic Optimism	−.39	***	.07		.09		.08	
Subjective norms	.56	***	.31	***	.31	***	.30	***
Descriptive norms	.32	***	.08		.07		.06	
Moral norms	.30	***	.07		.08		.08	
**R** ^2^			.39	***				
Stereotypical beliefs: Number of partners	.20	**			.04		.02	
Stereotypical beliefs: p x c	.13	*			−.04		−.05	
∆ Stereotypical beliefs: Number of partners	−.15	*			.06		.10	
**R** ^2^					.40	***		
New partner last 6 months	.39	***					.00	
Multiple partners last 6 months	.34	***					.12	*
**R** ^2^							.41	***

* Only predictors with a significant correlation with intention, p < .05

At T1, all social-cognitive determinants correlated significantly with intention, except perceived behavioral control. Intercorrelations for these determinants varied between .56 and .24. The variables in the regression equation together explained 39 % of the variance in intentions, but only susceptibility and subjective norms explained significant proportions of unique variance. Three stereotypical beliefs measures correlated with intention to test, but these correlations were relatively small. Adding these three stereotypical beliefs measures to the regression in step 2 barely increased the percentage of explained variance to 40 %; none of the variables made a significant unique contribution. Having had a new partner in the last 6 months, as well as having had multiple partners in the last six months, both positively correlated with intentions. Again, adding these two measures to the regression analysis in step 3 barely increased the percentage of explained variance in intentions to 41 %. In the final model, only the variables susceptibility, subjective norm, and having had multiple partners in the last 6 months made a significant unique contribution to the multivariate explanation of variance in intention. Overall, the percentage of explained variance did not substantially improve.

## Discussion

In this study, we found a substantial relationship between Ct testing intentions and subsequent testing behavior. Participants who expressed a higher intention to test at T0 indeed tested more often at T1. Those participants who had reported a low intention to test at T0 but that did test between T0 and T1 reported having participated in risky behavior during the 6 months in between. A large group of participants, who reported low intention to test, did not test and did not participate in high-risk behavior in the time between T0 and T1. Nevertheless, there was a substantial group of people who did report risky behavior, but who still indicated low intention to test and who did not subsequently test. Measures at T0 of implicit associations about testing (reassuring, threatening, annoying) were not related to testing behavior at T1. We found that the higher the participants’ own numbers of partners ever, the higher they estimated the number of partners of the stereotypical person with Ct. On the one hand, these stereotypical beliefs can be seen to reflect an underestimation of own risk, while on the other hand, the participant’s own behavior seems to provide a reason for testing. A logistic regression analysis showed testing behavior was preceded by high testing intentions and associated with having had multiple partners in the last six months, but could not be predicted with high accuracy.

Concerning the second research question, the prediction of testing intentions, Ct testing intentions at T1 can be predicted from the (explicit) determinants (step 1), stereotypical beliefs measures (step 2), and changes in partner status (step 3), all measured at T1. The *R* ^2^ indices for the three successive steps are .39, .40 and .41, which reflect large effect sizes, but also indicate that there was no relevant improvement in the percentage of explained variance in intentions with the addition of extra variables. In this study, susceptibility and subjective norms were the most influential explicit determinants. Reported risk behavior also appears to influence intentions, but only when risk behavior is related to the number of partners and not to the frequency of condom use. Measures of stereotypical beliefs did not provide relevant additional explanations for intentions to test. Participants’ own risk behaviors seem to be reflected in their susceptibility, but at the same time systematically underestimated. One reason for this might be that the higher the participants’ own numbers of partners ever, the higher they estimated the number of partners of the stereotypical person with Ct. Susceptibility for Ct seems to be derived more from the stereotypical person with Ct’s number of partners than with lack of condom use.

### Implications for interventions

Testing behavior appears to be influenced by testing intentions, which vary as a result of both changes in partner status and condom use. Intentions are, in turn, influenced by determinants, most strongly by susceptibility and subjective norms. However, other social-cognitive determinants are also significantly correlated with intention, some of which may be easier to change. Regression analyses can identify the best predictors of behavior, while correlations between determinants and intentions can provide the health educator with additional information about which determinants and underlying beliefs to target in an intervention [9, 10].

Our results also show that testing behaviors and intentions are related to own risk behavior (i.e., number of partners and having a new partner), but there is also a systematic tendency to underestimate own risk, especially the risk associated with inconsistent condom use. In terms of health education messages, it should not only be stressed that with every new partner there is a risk of infection, but also that there are other risks associated with getting infected with Ct, such as the inconsistent use of condoms, as well as having multiple partners.

This study confirms the results of earlier studies identifying relevant determinants of behavior: risk perception [11], perceived norms [12], stereotypical beliefs [6], and risk behavior [1]. At the same time, we see a general underestimation of own risk as compared to figures reported by other authors [13]. Any health promotion intervention directed at the general population will probably have a limited effect on testing behavior in terms of both percentages tested and tested positive. There are too many people who do not perceive their behavior as risky and who will not see any need for Ct testing. For many of them this may be an accurate assessment, but for too many of them it is an incorrect assessment [13]. As a result, any approach targeting the general population is not likely to result in a sufficient amount of participation, and will not, therefore, be cost-effective [3].

Making people more aware of their own risk is difficult, in part due to the defense mechanisms they may employ [14]. However, it has also been shown that applying threatening health messages is certainly not an effective behavior change method [4]. Some potentially promising alternatives that could be used to reach people at risk for Ct infection have been suggested in the literature. Online interventions [15] and screening in primary care [16] may go some way towards increasing the number of people participating in screening programs. Focusing on rescreening positives [17] and clinic-based interventions [18] would fit well with our finding regarding the relevance of susceptibility. Making testing easier and reducing barriers [19, 20] would be consistent with our findings on self-efficacy; while using social and sexual networks, for example to contact peers who have shown evidence of risky behavior, testing behavior, and Ct positivity [21], would be consistent with our findings on the relevance of both susceptibility and perceived norms.

### Limitations

One of the limitations of this study is that the sample was not representative of the age group, because high-risk participants were oversampled. Moreover, all participants had already participated in an earlier study. However, because we were interested in the relationships between measured determinants, intentions and behaviors, representativeness was not so relevant to our particular research design. A further limitation is that, overall, the number of people who test for Ct is low, which means that any comparisons between groups are often restricted by power issues. Moreover, all our T1 measures reflected behaviors and cognitions that took place during the last six months but we did not gather information about the timescale or order of events within this 6-month period. For example, we do not know if people tested before or after they decided not to use condoms. A last limitation may be that certain relevant determinants are still missing. Lessons were learned from our two earlier studies [1, 2], and some new determinants were measured in the present study. We found that these determinants were indeed related to testing intentions; however, the percentage of explained variance in intentions did not increase from that reported in our earlier studies. It is possible that an individual’s testing behavior is dependent on their partner’s intentions and behaviors and we were not able to measure these. Another possible determinant that we did not include in our study is stigma [22, 23]. Stigma might lead to lack of communication about Ct testing. We know that norms predict intention but stigma might limit the effects of interventions designed to increase communication about subjective and descriptive norm. Theunissen et al. [24] showed that people avoid stigmatizing reactions by limiting communication about Ct testing to their trusted network. Finally, Booth et al. [25, 26] have argued that stereotypical belief related questions should be asked about a person who tests for Ct instead of a person with Ct. Booth et al. also found self-identity to be a determinant of testing intentions.

## Conclusions

Most people who test for Ct are doing so for good reasons. Of course, adequate treatment of Ct positivity is the public health outcome needed in order to stop Ct transmission and prevent individual complications. There are people who are at risk for Ct infection but who do not intend to test, despite the fact that there do not seem to be many practical barriers to Ct testing (the studies thus far show that self-efficacy is high in all groups). In this study, we examined the role of a number of additional determinants for testing; however, their unique contribution to the explanation of intentions and behavior was non-existent. The most relevant determinants for testing intentions and testing behavior are perceived norms, susceptibility and changes in partner status. On the one hand, susceptibility seems to be based on an accurate interpretation of own risk behavior, number of partners and having a new partner, but on the other hand there is a systematic tendency to underestimate own risk. A general population approach to promote Ct testing will probably not be cost-effective. Future research should focus on more promising alternatives in order to promote testing behavior through existing communication channels.

## Additional file

Additional file 1:
**Database, syntax, and additional information for the study "Predictors of Chlamydia Trachomatis testing: perceived norms, susceptibility, changes in partner status, and underestimation of own risk".** (ZIP 104 kb)

## Abbreviations

Ct: c*hlamydia trachomatis*
