# Supplementary material for: Predictors of Chlamydia Trachomatis testing: perceived norms, susceptibility, changes in partner status, and underestimation of own risk
Source: BMC Public Health. 2016 Jan 20;16:55. doi: 10.1186/s12889-016-2689-6 (PMC4719691; doi:10.1186/s12889-016-2689-6)
Supplement: Additional file 1: — Database, syntax, and additional information for the study "Predictors of Chlamydia Trachomatis testing: perceived norms, susceptibility, changes in partner status, and underestimation of own risk". (ZIP 104 kb) [file 12889_2016_2689_MOESM1_ESM.zip › C3 Figure A tree.pptx]

## Slide 1
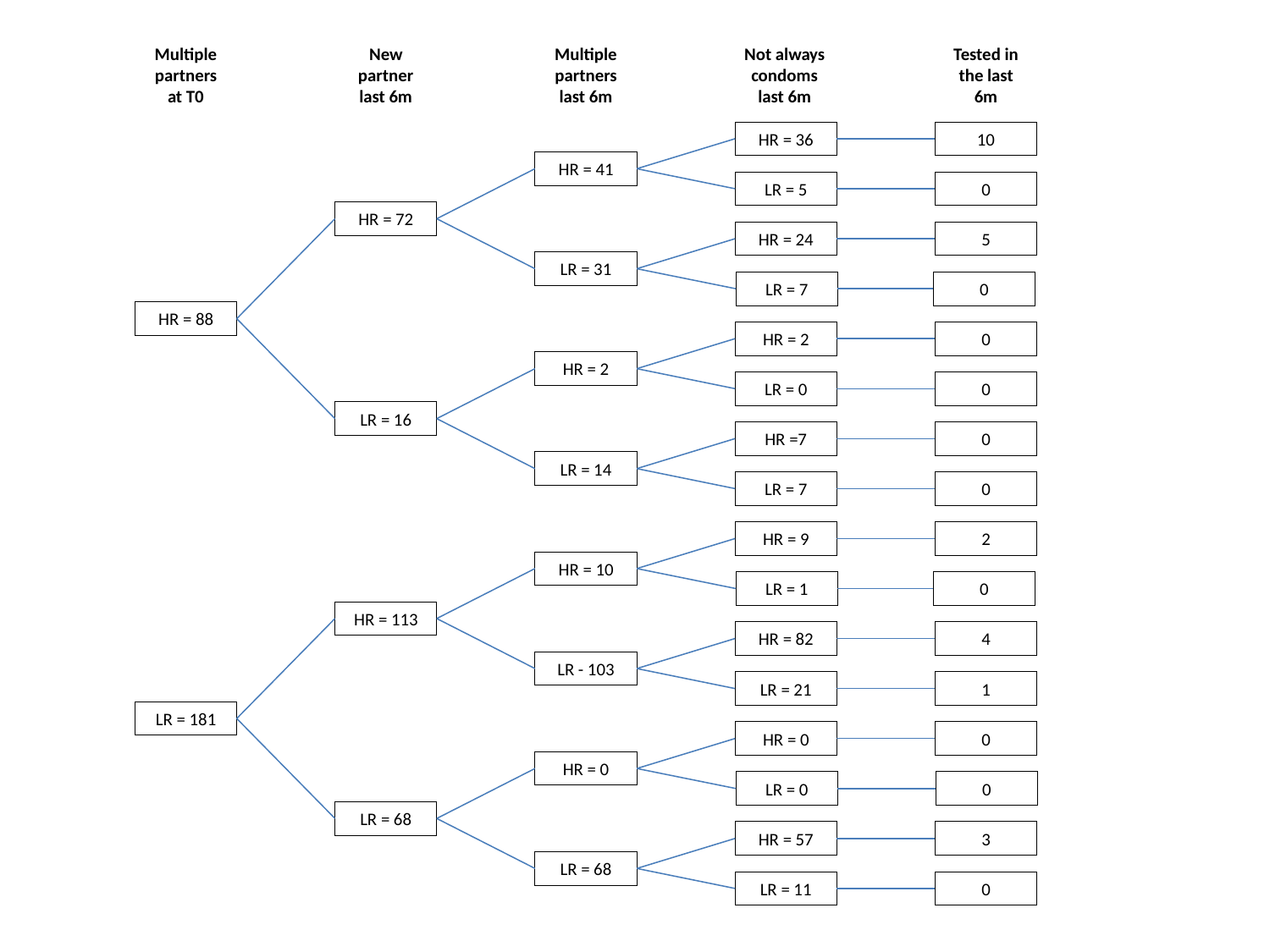

Multiple partners at T0
New partner last 6m
Multiple partners last 6m
Not always condoms last 6m
Tested in the last 6m
HR = 36
10
HR = 41
LR = 5
0
HR = 72
HR = 24
5
LR = 31
LR = 7
0
HR = 88
HR = 2
0
HR = 2
LR = 0
0
LR = 16
HR =7
0
LR = 14
LR = 7
0
HR = 9
2
HR = 10
LR = 1
0
HR = 113
HR = 82
4
LR - 103
LR = 21
1
LR = 181
HR = 0
0
HR = 0
LR = 0
0
LR = 68
HR = 57
3
LR = 68
LR = 11
0
